# Magnetohydrodynamics flow of a nanofluid driven by a stretching/shrinking sheet with suction

**DOI:** 10.1186/s40064-016-3588-0

**Published:** 2016-11-02

**Authors:** U. S. Mahabaleshwar, P. N. Vinay Kumar, Mikhail Sheremet

**Affiliations:** 1 Department of Mathematics, Government First Grade College for Women, Hassan, Karnataka 573 201 India; 2 Department of Mathematics, SHDD Government First Grade College, Paduvalahippe, Hassan, Karnataka 573 201 India; 3 Department of Theoretical Mechanics, Tomsk State University, 36 Lenin Avenue, Tomsk, 634050 Russia

**Keywords:** Non-linear differential equation, Nanofluid, MHD stretching/shrinking sheet, Suction/injection

## Abstract

The present paper investigates the effect of a mathematical model describing the aforementioned process in which the ambient nanofluid in the presence of suction/injection and magnetic field are taken into consideration. The flow is induced by an infinite elastic sheet which is stretched along its own plane. The stretching/shrinking of the sheet is assumed to be proportional to the distance from the slit. The governing equations are reduced to a nonlinear ordinary differential equation by means of similarity transformation. The consequential nonlinear equation is solved analytically. Consequences show that the flow field can be divided into a near-field region and a far-field region. Suction on the surface plays an important role in the flow development in the near-field whereas the far-field is responsible mainly by stretching. The electromagnetic effect plays exactly the same role as the MHD, which is to reduce the horizontal flow resulting from stretching. It is shown that the behavior of the fluid flow changes with the change of the nanoparticles type. The present study throws light on the analytical solution of a class of laminar boundary layer equations arising in the stretching/shrinking sheet problem.

## Background

Dynamics of fluid flow over a linear stretching/shrinking sheet plays very significant role in many manufacturing applications. The thin polymer sheet constitutes a continuously moving solid surface with a non-uniform surface velocity through an or else quiescent fluid. The cooling fluids in past times was selected to be the in large quantities available water, but this has the disadvantage of speedily quenching the heat leading to rapid solidification of the stretching sheet (see Andersson 1995, 2002, Fisher 1976, Siddheshwar and Mahabaleshwar 2005). From the standpoint of desirable properties of the final product water does not seem to be the ideal cooling fluid.

The word of nanofluid refers to a solid–liquid mixture with a continuous phase which is a nanometer sized nanoparticle dispersed in conventional base liquids. Nanofluids are base-fluids containing suspended nanoparticles. These nanoparticles are typically mad of metals, oxides, or carbon nanotubes. There are a few well-known correlations for predicting the thermal and physical properties of nanofluids which are often cited by researchers to calculate the convective heat transfer behaviors of the nanofluids. The word “nanofluid” coined by Choi (1995) describes a liquid suspension containing ultra-fine particles (diameter less than 50 nm) (See Choi et al. 2001, Keblinski et al. 2005). The first providing for this ground of Sakiadis (1961a, b, c) he concentrated on the induced affected by the uniform motion of a continuous solid surface taking into account the laminar boundary layer approximation.

An exact analytical solution of the equation for a elastic sheet where the surface stretching velocity was proportional to the distance from the slot was given in Crane (1970). In this presentation, we will perform an analysis of a mathematical model describing the aforementioned process in which the ambient nanofluid in the presence of mass transfer is taken into consideration.

## Solution of mathematical formulation

**Fig. 1:**
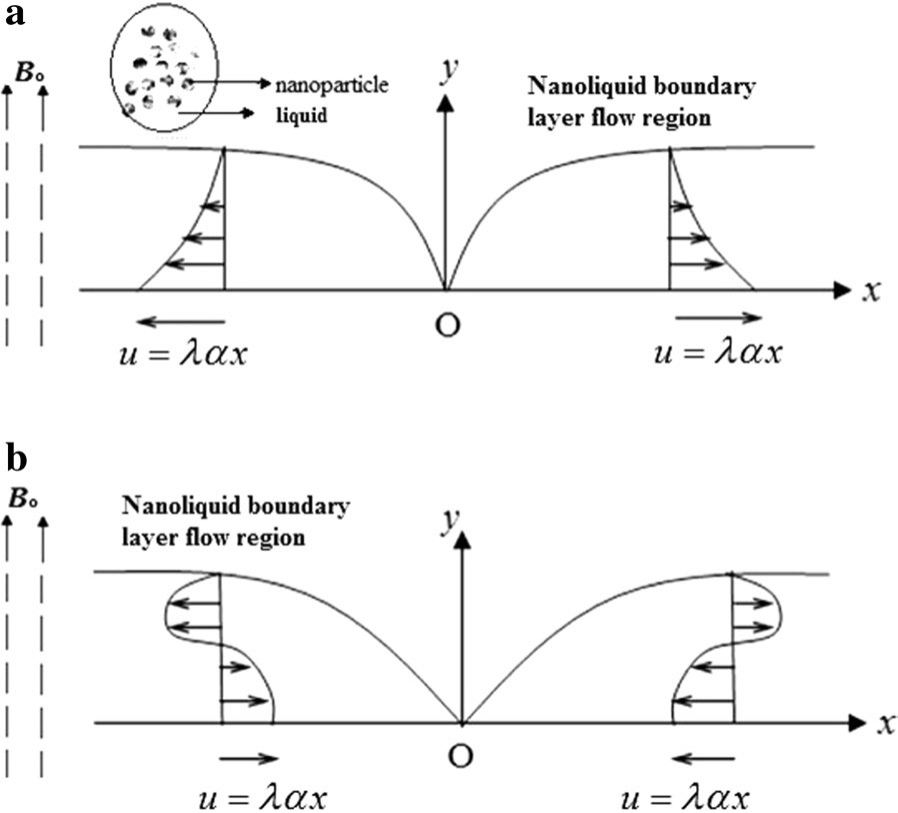
The schematic flow diagram of stretching/shrinking boundary **a** Stretching sheet case (*λ* > 0) **b** Shrinking sheet case (*λ* < 0)

We reflect on the steady laminar boundary two-dimensional (*x*, *y*) co-ordinate magnetohydrodynamic (MHD) flows of a nanofluid past stretching/shrinking sheet in the presence of presence of mass transfer. The liquid is electrically conducting in the presence of applied magnetic field with constant strength *B*_0_ that is parallel to *y*-axis. The sheet is supposed extended in the *x*-direction such that the *x*-component of velocity varies linearly with *x* along its surface. It is assumed that the velocity distribution of the stretching/shrinking sheet is $u = \lambda u_{w} (x) = \lambda \alpha x$ where *x* is the coordinates calculated along the stretching/shrinking of the sheet, $\lambda$ is a constant with $\lambda > 0$ for a stretching, $\lambda < 0$ for a shrinking and $\lambda = 0$ surface is permeable. Stretching/shrinking sheet problems Prandtl zero pressure gradient and outside other forces are not considered. In practice, it is only an extremely meticulous pulling of the sheet that can allow one to assume linear stretching. The schematic of the physical replica, geometrical coordinates are depicted in Fig. 1. The liquid is a based nanoliquids containing three types namely, Cu, Al_2_O_3_ and TiO_2_. Thermophysical properties of the nanoliquid are listed in the below Table 1.

**Table 1:** Thermophysical properties of the base fluid (water) and nanoparticles

Nanoliquid physical properties	Liquid phase (water)	Copper	Alumina	Titania
*C*_*p*_ (J/kg K)	4179	385	765	686.2
$\rho$ (kg/m^3^)	997.1	8933	3970	42.50
*k* (W/m K)	0.613	400	40	8.9538

Conservation of mass and conservation linear momentum are given by

1 $$\frac{{\partial q_{i} }}{{\partial x_{i} }} = 0,$$

2 $$\rho_{nf} \left[ {\frac{{\partial q_{i} }}{\partial t} + q_{j} \frac{{\partial q_{i} }}{{\partial x_{j} }}} \right] = - \frac{\partial p}{{\partial x_{i} }} + \mu_{nf} \nabla^{2} q_{i} - \sigma B_{o}^{2} q_{i} ,$$

where, $\frac{d}{dt} = \frac{\partial }{\partial t} + q_{j} \frac{\partial }{{\partial x_{j} }}$, where, the other quantities have their meaning as mentioned in nomenclature.

The bases for present analysis laminar boundary layer equations for an incompressible nanofluid.

3 $$\frac{\partial u}{\partial x} + \frac{\partial v}{\partial y} = 0,$$

4 $$u\frac{\partial u}{\partial x} + v\frac{\partial u}{\partial y} = \frac{{\mu_{{_{nf} }} }}{{\rho_{nf} }}\frac{{\partial^{2} u}}{{\partial y^{2} }} - \frac{{\sigma B_{0}^{2} }}{{\rho_{nf} }}u,$$

where, the quantities have their meaning as mentioned in nomenclature. We further assume *R*_m_ ≪ 1, where *R*_m_ is the magnetic Reynolds number.

 The associated boundary conditions on velocity are given by

5a $${u = \lambda u_{w} (x) = \lambda \alpha x,} \quad {{\text{v}} = {\text{v}}_{c} ,} \quad {{\text{at}}\, y = 0,}$$

5b $${u \to 0,}\quad {{\text{as}}\quad y \to \infty .}$$

Laminar boundary layer flows induced by a continuous surface stretching with velocity $u_{w} (x)$, $v_{c}$ is the mass flux velocity with $v_{c} < 0$ for suction, $v_{c} > 0$ for injection and $v_{c} = 0$ is the case when the surface is impermeable.

In the physical stream function formulation $\psi \left( {x,y} \right)$ such that

6 $$u = \frac{\partial \psi }{\partial y}\quad {\text{and}}\quad v = - \frac{\partial \psi }{\partial x},$$

where, $\psi \left( {x,y} \right) = \lambda \sqrt {\nu_{f} } x \, f\left( \eta \right),$ $\, f\left( \eta \right)$ is the dimensionless stream function and $\eta = \left( {\sqrt {\frac{\alpha }{{\nu_{f} }}} } \right)y$. The material parameters in (4) are described mathematically by,

$$\mu_{nf} = \frac{{\mu_{f} }}{{\left( {1 - \phi } \right)^{2.5} }}\quad {\text{and}}\quad \rho_{nf} = \rho_{f} \left( {1 - \phi } \right) + \rho_{s} \phi ,$$

where, $\phi$ ($0 < \phi < 1$) is the solid volume fraction, $\rho_{s}$ is for nanosolid-particles, $\rho_{f}$ is for base fluid.

Equations (3) and (4) admit self-similar solution of the appearance

7 $$u = \alpha x\,f_{\eta },\quad v = - \sqrt {\alpha \,\nu_{f} } \,f\left( \eta \right),$$

where, subscript $\eta$ denotes the derivative.

In the stream function formulation Eq. (6), Eqs. (3) and (4) reduce to

8 $$\frac{{\partial^{3} \psi }}{{\partial y^{3} }} + \frac{{\rho_{nf} }}{{\mu_{nf} }}\frac{{\partial \left( {\psi ,\frac{\partial \psi }{\partial y}} \right)}}{{\partial \left( {x,y} \right)}} - \frac{{\sigma B_{0}^{2} }}{{\mu_{nf} }}\frac{\partial \psi }{\partial y} = 0,$$

where the second term in the above equation is the Jacobian. Substituting $\psi \left( {x,y} \right) = \lambda \sqrt {\nu_{f} } x \, f\left( \eta \right),$ into Eq. (8) and following ordinary differential equation

9a $$f_{\eta \eta \eta } + \left( {1 - \phi } \right)^{2.5} \left\{ {\left( {1 - \phi + \frac{{\rho_{s} }}{{\rho_{f} }}\phi } \right)\left\{ {f\,f_{\eta \eta } - \left( {f_{\eta } } \right)^{2} } \right\} - \frac{{\sigma B_{0}^{2} }}{{\alpha \,\rho_{{_{f} }} }}f_{\eta } } \right\} = 0,$$

This can be rewritten as,

9b $$f_{\eta \eta \eta } + \Gamma_{1} \left\{ {\Gamma_{2} \left\{ {f\,f_{\eta \eta } - \left( {f_{\eta } } \right)^{2} } \right\} - Qf_{\eta } } \right\} = 0,$$

where, $\Gamma _{1} = \left( {1 - \phi } \right)^{2.5}$, $\Gamma_{2} = 1 - \phi + \frac{{\rho_{s} }}{{\rho_{f} }}\phi$ and $Q = \frac{{\sigma B_{0}^{2} }}{{\alpha \,\rho_{{_{f} }} }}$ is the Chandrasekhar number ($\sqrt {Q\,}$ is called Hartmann number).

The associated boundary conditions are given by

10 $$f\left( 0 \right) = V_{c} ,\quad f_{n} \left( 0 \right) = \lambda ,\quad f^{\prime}\left( \infty \right) \to 0,$$

where, $V_{c} = \frac{{v_{c} \,}}{{\sqrt {\alpha \,\nu_{f} } }}$ suction/injection, $\lambda$ represents stretching/shrinking parameter, $\lambda > 0$ represents stretching sheet, $\lambda < 0$ represents shrinking sheet and $\lambda = 0$ for fixed surface.

We search the solution of the laminar boundary value problem (9) and (10) in the following closed analytical form,

11 $$f\left( \eta \right) = V_{C} + \lambda \left[ {\frac{1 - Exp( - \beta \eta )}{\beta }} \right],$$

where, $\beta > 0$ must satisfy the quadratic equation,

12 $$\zeta^{2} - \Gamma_{1} \Gamma_{2} V_{c} \zeta - \Gamma_{1} \left( {\Gamma_{2} \lambda + Q} \right) = 0,$$

13 $$\beta = \frac{{\Gamma_{1} \Gamma_{2} V_{c} \pm \sqrt {\left( {\Gamma_{1} \Gamma_{2} V_{c} } \right)^{2} + 4\Gamma_{1} \left( {\Gamma_{2} \lambda + Q} \right)} }}{2},$$

The discriminant of Eq. (13) is positive when $\lambda \ge 0$ and it can be negative, if $\lambda < 0$. In the latter case the discriminant is non-negative only if

13a $$\lambda \ge \frac{{\left( {\Gamma_{1} \Gamma_{2} V_{c} } \right)^{2} }}{4} - \frac{Q}{{\Gamma_{2} }}.$$

So in the case of a shrinking sheet if the inequality (13a) is not satisfied it is impossible to find $\beta$ and the analytical solution of the required form does not exist.

Suppose now that the discriminant is ≥0 and distinguish some cases.

**Case (i)**: If *V*_*c*_ = 0 and $\Gamma_{2} \lambda + Q > 0,$ then $\beta = \Gamma_{1} \sqrt {\Gamma_{2} \lambda + Q}$, if *V*_*c*_ = 0 and $\Gamma_{2} \lambda + Q \le 0$ it is not possible to find $\beta$.

**Case (ii)**: If $V_{c} \ne 0$ and $\Gamma_{2} \lambda + Q > 0,$ then the quadratic equation admits two roots of different sign and

13b $$\beta = \frac{{\Gamma_{1} \Gamma_{2} V_{c} \pm \sqrt {\left( {\Gamma_{1} \Gamma_{2} V_{c} } \right)^{2} + 4\Gamma_{1} \left( {\Gamma_{2} \lambda + Q} \right)} }}{2}.$$

If $V_{c} \ne 0$ and $\Gamma_{2} \lambda + Q < 0,$ then the quadratic equation admits two roots of the same sign; if $V_{c} > 0$ the two roots are positive and so $\beta$ has two possible values:

13c $$\frac{{\Gamma_{1} \Gamma_{2} V_{c} + \sqrt {\left( {\Gamma_{1} \Gamma_{2} V_{c} } \right)^{2} + 4\Gamma_{1} \left( {\Gamma_{2} \lambda + Q} \right)} }}{2}$$

and

13d $$\frac{{\Gamma_{1} \Gamma_{2} V_{c} - \sqrt {\left( {\Gamma_{1} \Gamma_{2} V_{c} } \right)^{2} + 4\Gamma_{1} \left( {\Gamma_{2} \lambda + Q} \right)} }}{2}.$$

Therefore in this case the problem (9) and (10) admits two analytical solutions. If $V_{c} < 0$, the two roots are negative and so the problem does not admit a solution in closed form. If $\Gamma_{2} \lambda + Q = 0,$ and $V_{c} > 0$, then $\beta = \Gamma_{1} \Gamma_{2} V_{c}$; if $\Gamma_{2} \lambda + Q = 0$ and $V_{c} < 0$, then it is impossible to find $\beta$.

Finally if $\lambda < 0,$ and the discriminant is equal to 0, then in the case $V_{c} > 0$ we have $\beta = \frac{{\Gamma_{1} \Gamma_{2} V_{c} }}{2}$, while in the case $V_{c} < 0$ it is impossible to find $\beta$. When it is impossible to find $\beta$ one can try to solve the problem numerically. More over the possibility of two values of $\beta$ is not surprising because in the studies on the flows of the classical fluids with a stretching/shrinking sheet dual solutions have been found in the literature.

### Skin friction

Wall shearing stress $\tau_{w}$ the expression is given by:

14 $$\tau_{w} = \, - \mu_{nf} \,\left( {\frac{\partial \,u}{\partial \,y}} \right)_{y\, = \,0} = - \frac{1}{{\left( {1 - \phi } \right)^{2.5} }}\rho_{f} \sqrt {\nu_{f} \alpha^{3} } x\,f_{\eta \eta } \left( 0 \right).$$

Substituting $u = \alpha \,x\,\lambda e^{ - \beta \eta }$ in Eq. (14), we get

15 $$\tau_{w} = \mu_{nf} \,\alpha^{3} \,x\,\beta \,\sqrt {\frac{\lambda }{{\nu_{f} }}} .$$

## Results and discussion

The present article is the generalization of the classical work of Crane (1970) flow and nanofluid driven by stretching/shrinking sheet with external magnetic field and suction. The classical Crane solution of the linear stretching sheet is extensive to include nanofluid, shrinking and suction/injection of weakly electrically conducting Newtonian fluids and also three types nanofluids, namely Copper (Cu), alumina (Al_2_O_3_) and Titania (TiO_2_) in water as the base fluid. The basic boundary layer equation of momentum field is mapped into highly nonlinear ordinary differential equations via similarity transformations. Similarity solution is obtained for the velocity distribution. The velocities are decreasing function of $\eta$ as it is an exponential function with negative argument. It is apparent from Eq. (11), that is $\beta$, which is function of the suction/injection parameter $V_{c}$, with $V_{c} < 0$ for suction, $V_{c} > 0$ for injection and $V_{c} = 0$ is the case when the surface is impermeable, stretching/shrinking parameter $\lambda$, $\lambda > 0$ for stretching sheet, $\lambda < 0$ for a shrinking and $\lambda = 0$ for fixed surface and Chandrasekhar number *Q*, shows to the slope of above exponentially decreasing velocity profiles.

**Fig. 2:**
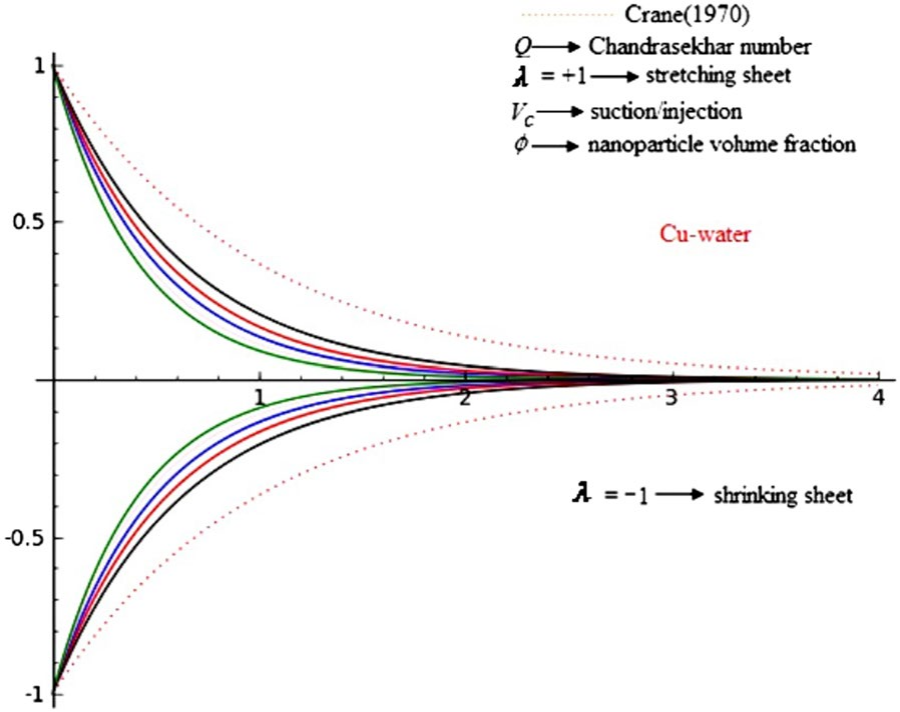
Effects of Chandrasekhar number *Q* on axial $f_{\eta }$ velocity in the case of Copper (Cu)-water with $\phi = 0.2$ and $V_{c} = 0.4$

**Fig. 3:**
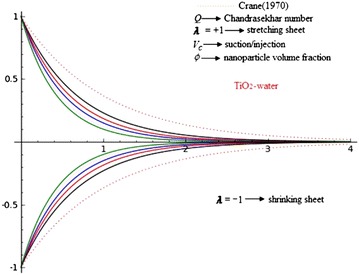
Effects of Chandrasekhar number *Q* on axial $f_{\eta }$ velocity in the case of Alumina (Al_2_O_3_)-water with $\phi = 0.2$ and $V_{c} = 0.4$

**Fig. 4:**
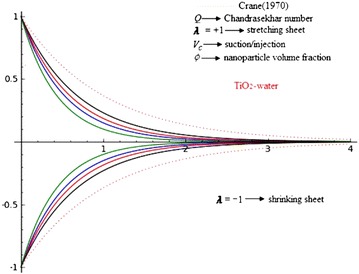
Effects of Chandrasekhar number *Q* on axial $f_{\eta }$ velocity in the case of Titania (TiO_2_)-water with $\phi = 0.2$ and $V_{c} = 0.4$

Figures 2, 3 and 4 reveals the influences of Chandrasekhar number *Q*, on the laminar boundary layer flow field. The presence of Chandrasekhar number *Q* sets in Lorentz force effect, which consequences in the retarding effect on the velocity field. As the values of Chandrasekhar number *Q*, increase, the retarding force increases and consequently the velocity decreases. The same effect is observed for increasing values of $V_{c} > 0$, it is also clear that increasing values of *Q* results in flattening of $f_{\eta }$. These figures reveals that velocity profiles are going closer to the wall and the boundary layer thickness becomes thinner for the increasing *Q*. It is seen that the velocity is going closer to the wall and boundary layer thickness becomes thinner for larger *Q*. The reason behind this is that increase in *Q* results the increase in Lorentz force which in turn produce more resistance to the velocity field. Physically, present phenomena occur when magnetic field can induced current in the conductive fluid, then it create a resistive-type force on the fluid in the boundary layer, which slow down the motion of the fluid. So finally, it is conclude that magnetic field is used to control boundary layer separation. The thickness of MHD boundary layer also depends upon the $\lambda$. For $\lambda = - 1$, the laminar boundary layer thickness is larger than a $\lambda = + 1$ and the effects of *Q* are more pronounced. These effects are negligible for $\lambda = 1$.

## Concluding remarks

The laminar boundary layer flows in a nanofluid induced as a result of motion of a stretching/shrinking sheet has been presented. We study only analytical solution of the problem and some important results of the study are concluded as follows:

1. The axial velocity and transverse velocity, is a decreasing function of $\eta$ as it is an exponential function with negative argument.
2. Increasing values of the *Q* results in pulling down of velocity profiles.
3. Velocity profiles decrease with an increase in *Q* (Ferraro and Plumpton 1961) and (Borrelli et al. 2015).
4. The velocity components transverse velocity $f$ and axial $f_{\eta }$ are reveals for different values of the *Q*, the velocity decreases with increases in the *Q* due to an increase in the Lorentz drag force that opposes the fluid motion.
5. The increase of *Q* leads to the increase of skin friction parameter in all the cases of suction/injection.
6. The classical Crane (1970) flow is recovered from Eq. (13) for $V_{c} = Q = \phi = 0$ and $\lambda = \Gamma_{1} = \Gamma_{2} = 1$.
7. The classical Pavlov (1974) flow is recovered from Eq. (13) for $V_{c} = \phi = 0$ and $\lambda = \Gamma_{1} = \Gamma_{2} = 1$.
8. The Gupta and Gupta (1977) flow is recovered from Eq. (13) for $\phi = 0$ and $\lambda = \Gamma_{1} = \Gamma_{2} = 1$.
9. The skin friction is lower for stretching and higher for shrinking sheets.
10. The effect of increasing the $V_{c}$ and the *Q* is to increase the velocity and decrease the laminar boundary layer thickness in shrinking case (Borrelli et al. 2012, 2013a, b).
11. The heat transfer at the surface of the sheet increases with the increasing suction/injection $V_{c}$ and the nanoparticle solid volume fraction $\phi$.

## List of symbols

*C*_*f*_: skin friction coefficient
*B*_0_: magnetic field (w m^−2)^
*f*: dimensionless stream function
***J***: current density
*q*_*i*_ and *q*_*j*_: velocity components
$$\text{Re}_{x}$$
local Reynolds number $\left( {\text{Re}_{x} = \frac{{xu_{w} }}{{\nu_{f} }}} \right)$
*u*: axial velocity part along *x*-axis (m s^−1^)
*v*: transverse velocity part along *y*-axis (m s^−1^)
$$V_{c}$$
constant suction/injection parameter $V_{C} = \frac{{v_{c} }}{{\sqrt {\alpha \,v_{f} } }}$
*x*: horizontal coordinate (m)
*y*: vertical coordinate (m)

### Greek symbols

*α*: constant in the sheet coefficient (s^−1^), $(\alpha > 0)$
$$\lambda$$
constant, represents stretching/shrinking parameter
$$\eta \,$$
similarity variable = $$\left( {\sqrt {\frac{\alpha }{{\nu_{f} }}} } \right)y$$
$$\mu_{nf}$$
viscosity of the nanofluid (kg m^−1^ s^−1^)
$$\nu_{f}$$
kinematic viscosity of the fluid (m^2^ s^−1^)
$$\rho_{nf}$$
density of the nanofluid (kg m^−3^)
$$\rho_{f}$$
density of the fluid (kg m^−3^)
$$\rho_{s}$$
density of the nanosolid particles
$$\sigma$$
electrical conductivity of fluid (mho m^−1^)
$$\tau_{w}$$
wall shearing stress (m^2^ s^−1^)
$$\phi$$
nanoparticle volume fraction
$$\psi$$
physical stream function (m^2^ s^−1^)

### Subscripts/superscripts

0: origin
*f*: fluid
s: solid
*w*: wall condition
$$\infty$$
for from the sheet
$$f_{\eta }$$
first derivative w.r. t. $$\eta$$
$$f_{\eta \eta }$$
second derivative w.r. t. $$\eta$$
$$f_{\eta \eta \eta }$$
third derivative w.r. t. $$\eta$$
